# A case report of individualized ventilation in a COVID-19 patient – new possibilities and caveats to consider with flow-controlled ventilation

**DOI:** 10.1186/s12871-021-01365-y

**Published:** 2021-05-12

**Authors:** Patrick Spraider, Gabriel Putzer, Robert Breitkopf, Julia Abram, Simon Mathis, Bernhard Glodny, Judith Martini

**Affiliations:** 1 Department of Anaesthesiology and Intensive Care Medicine, Medical University Innsbruck, Innsbruck, Austria; 2 Department of Radiology, Medical University Innsbruck, Innsbruck, Austria

**Keywords:** Respiratory Distress Syndrome, Adult, COVID-19, Case Report, Lung Compliance, Stress Mechanical

## Abstract

**Background:**

Flow-controlled ventilation (FCV) is a novel ventilation method increasingly being used clinically, particularly during the current COVID-19 pandemic. However, the continuous flow pattern in FCV during inspiration and expiration has a significant impact on respiratory parameters and ventilatory settings compared to conventional ventilation modes. In addition, the constant flow combined with direct intratracheal pressure measurement allows determination of dynamic compliance and ventilation settings can be adjusted accordingly, reflecting a personalized ventilation approach.

**Case presentation:**

A 50-year old women with confirmed SARS-CoV-2 infection suffering from acute respiratory distress syndrome (ARDS) was admitted to a tertiary medical center. Initial ventilation occurred with best standard of care pressure-controlled ventilation (PCV) and was then switched to FCV, by adopting PCV ventilator settings. This led to an increase in oxygenation by 30 %. Subsequently, to reduce invasiveness of mechanical ventilation, FCV was individualized by dynamic compliance guided adjustment of both, positive end-expiratory pressure and peak pressure; this intervention reduced driving pressure from 18 to 12 cm H_2_O. However, after several hours, compliance further deteriorated which resulted in a tidal volume of only 4.7 ml/kg.

**Conclusions:**

An individualized FCV approach increased oxygenation parameters in a patient suffering from severe COVID-19 related ARDS. Direct intratracheal pressure measurements allow for determination of dynamic compliance and thus optimization of ventilator settings, thereby reducing applied and dissipated energy. However, although desirable, this personalized ventilation strategy may reach its limits when lung function is so severely impaired that patient’s oxygenation has to be ensured at the expense of lung protective ventilation concepts.

## Background

An inherent problem of artificial ventilation is the difficulty to precisely assess individual lung mechanics from ventilation parameters and displayed measurements, knowing that setting the ventilator based on body weight does not adequately address variations in lung mechanics. This fact is especially critical in severely ill patients such as patients suffering from SARS-CoV-2 associated acute respiratory distress syndrome (ARDS) [1–3]. Flow-controlled ventilation (FCV) is a novel ventilation mode which, due to its constant flow and direct intratracheal pressure measurement, enables accurate measurement of dynamic compliance. In the following article we would like to demonstrate by means of a case report how individualized ventilation with compliance guided pressure settings can be performed with FCV, thereby reducing the risk of ventilator induced lung injury on the one hand and improving ventilation efficiency on the other.

## Case Presentation

**Fig. 1:**
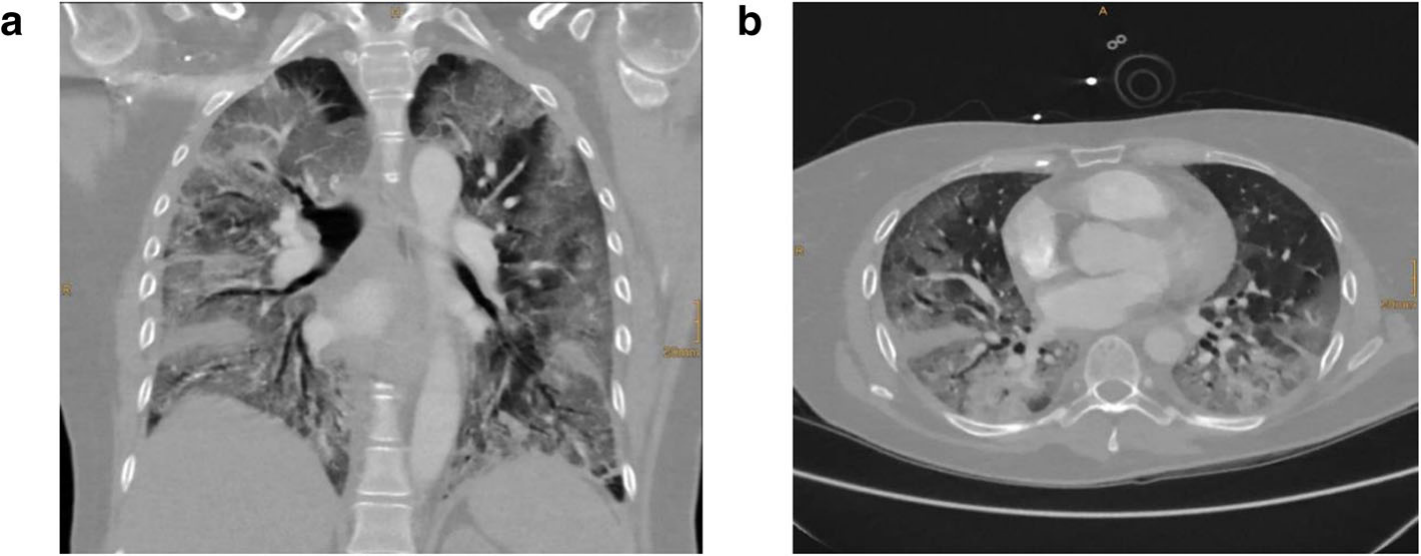
Computed tomography four days prior to admission showing spacious areas of ground glass opacities including consolidation and air bronchogram (**a**, coronal plane; **b**, axial plane, with kind permission of the Department of Radiodiagnostics, Central Hospital Bolzano, Bozen/Bolzano, Italy)

A 50-year-old woman with confirmed SARS-CoV-2 infection was admitted to a peripheral hospital three weeks prior to admission to our hospital. Because of deterioration of respiratory function, the patient had to be intubated and mechanically ventilated. A CT-scan was performed at this stage revealing spacious areas of ground glass opacities including consolidation and air bronchogram (Fig. 1). As the patient’s situation worsened, she was transferred to the University Hospital Innsbruck, a tertiary medical center, for evaluation of extracorporeal membrane oxygenation (ECMO) therapy.

Evaluation of the pulmonary state revealed a moderate, SARS-CoV-2-associated ARDS (paO_2_/FiO_2_ ratio of 124) according to Berlin definition [4]. Initially, pressure-controlled ventilation (PCV) was applied with an Evita® Infinity® V500 respirator (Dräger, Lübeck, Germany) with a peak inspiratory pressure (P_peak_) of 27 cm H_2_O, a positive end-expiratory pressure (PEEP) of 12 cm H_2_O, an inspiration to expiration ratio (I:E-ratio) of 1:1, and a respiratory rate of 21 /min, resulting in a tidal volume of 5.8 ml/kg predicted body weight (PBW) and a compliance of 24 ml/cm H_2_O.

In accordance with ELSO guidelines [5], ECMO therapy was declined. Prone position improved the paO_2_/FiO_2_ ratio to 130–150 and was performed for the next 16 h. After this period compliance worsened again in supine position and invasiveness of ventilation had to be increased (P_peak_ 28 cm H_2_O, PEEP 10 cm H_2_O, respiratory rate of 25 /min, calculated mechanical power (MP) [6] of 16.9 J/min). Nevertheless, oxygenation was still compromised with a paO_2_ of 78.2 mmHg (10.4 kPa) at an FiO_2_ of 0.6 (paO_2_/FiO_2_ ratio of 130).

In an attempt to reduce the invasiveness of ventilation and its deleterious effects on lung tissue flow-controlled ventilation (FCV) was initiated with the Evone® respirator (Ventinova Medical B.V., Eindhoven, The Netherlands). This mode of ventilation can be applied via any conventional endotracheal tube and establishes a continuous, constant flow during inspiration as well as expiration, which is monitored and controlled by direct intratracheal pressure measurement. First, identical PCV pressure settings were adopted to FCV with a P_peak_ of 28 cm H_2_O, a PEEP of 10 cm H_2_O, an I:E-ratio of 1:1 and the flow set to 12 l/min, otherwise deep sedation (Richmond Agitation-Sedation Scale of -4) was continued with midazolam 0.4–0.5 mg/kg/h, morphine 0.4 mg/kg/h and esketamine 2.1 mg/kg/h without the use of neuromuscular blocking agents.

**Fig. 2:**
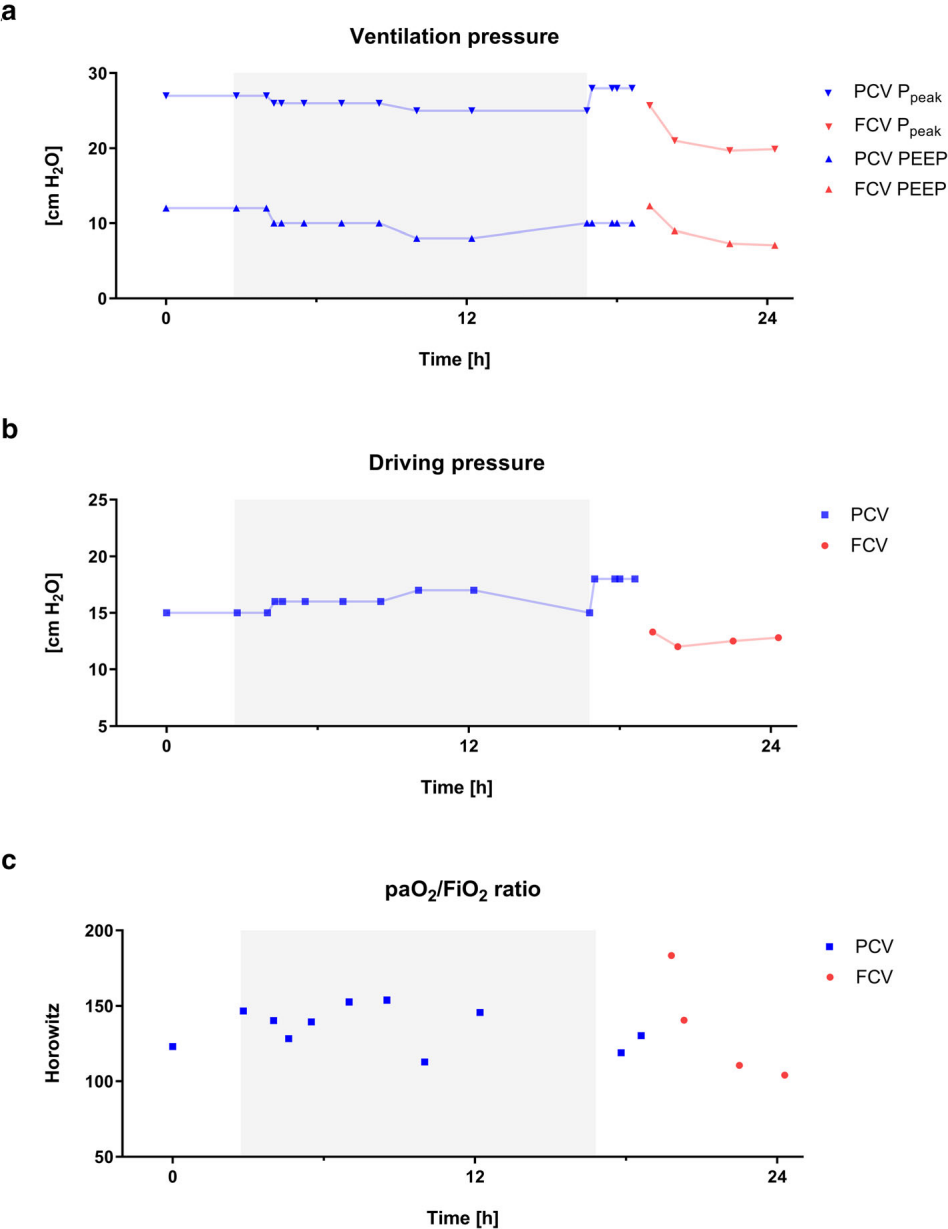
demonstrates the course of respiratory parameters during pressure-controlled ventilation (PCV; blue) and flow-controlled ventilation (FCV; red) since hospital admission. The grey area indicates prone position of the patient, the white area supine position. For FCV the (effective) alveolar pressures are calculated based on the measured resistance and the set flow according to Ohm’s law. **a**, peak inspiratory pressure (P_peak_) and positive end-expiratory pressure (PEEP). **b**, driving pressure. **c**, ratio of arterial partial pressure of oxygen (paO_2_) to fraction of inspired oxygen (FiO_2_)

The continuous, constant flow in FCV creates a continuous, stable pressure drop throughout inspiration and expiration from the set and measured tracheal pressure to the alveolar space and vice versa, as there is no pause during the ventilation cycle [7]. As a flow of gas only occurs if a pressure gradient exists, this gradient can be calculated if the flow and the resistance of the system are known. This relationship is based upon Ohm’s law, where the pressure drop (ΔP) equals the flow ($\dot{V}$) multiplicated by the resistance (R) of the system. Translated to the described patient, a pressure drop of 2.3 cm H_2_O between the trachea and the alveolar space can be calculated (ΔP = $\dot{V}$ x R; flow = 12 l/min = 0.2 l/s; resistance = 11.6 cm H_2_O/l/s). During inspiration when gas flow is directed to the alveolar space this fact reduced alveolar P_peak_ by 2.3 cm H_2_O (from 28 to 25.7 cm H_2_O), whereas during expiration when flow changes direction, PEEP was increased by 2.3 cm H_2_O (from 10 to 12.3 cm H_2_O). Therefore, driving pressure was reduced by 4.6 cm H_2_O compared to PCV (from 18 cm H_2_O to 13.4 cm H_2_O; Fig. 2b**).** In PCV however, airway pressure is presumed to be equal to alveolar pressure due to a zero flow phase at the end of inspiration as well as expiration, which should result in an equilibrium pressure phase.

**Fig. 3:**
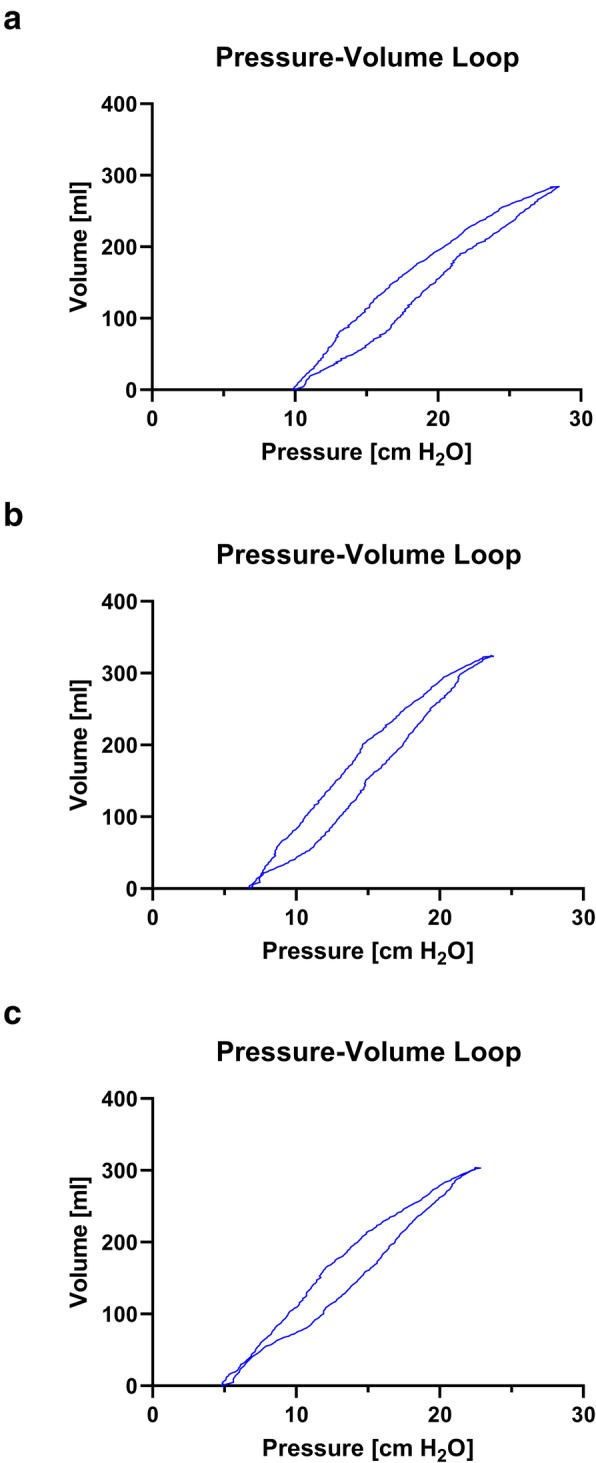
Pressure-volume loops obtained from intratracheal pressure measurement. **a**, represents the pressure-volume loop at the beginning of FCV with a peak inspiratory pressure (P_peak_) set to 28 cm H_2_O and a positive end-expiratory pressure (PEEP) of 10 cm H_2_0. Considering the measured resistance of 11.7 cm H_2_O/l/s and the set flow of 12 l/min and applying Ohm’s law, the alveolar P_peak_ calculates to only 25.7 cm H_2_O and the alveolar PEEP to 12.3 cm H_2_O. **b**, after compliance-guided pressure settings P_peak_ was reduced to 23 cm H_2_O (alveolar 21 cm H_2_O) and PEEP 7 cm H_2_O (alveolar 9 cm H_2_O) resulting in an almost linear relation of pressure and volume during in- and expiration. The steeper course of the PV loop in B compared to A indicates the increased compliance after individualized pressure settings. **c**, two hours after first compliance-guided pressure setting, re-evaluation showed a slight decline in lung mechanics. Therefore, P_peak_ was adjusted to 22 cm H_2_O (alveolar 20 cm H_2_O) and PEEP to 5 cm H_2_O (alveolar 7 cm H_2_O). The initial kinking of the inspiratory pressure volume curve in C is an indicator for intratidal recruitment

After 30 min, blood gas analysis revealed an improvement of oxygenation resulting in a paO_2_ of 110 mmHg (14.7 kPa) (paO_2_/FiO_2_ ratio of 183; Fig. 2c). In order to decrease invasiveness, ventilation settings were then adjusted to dynamic lung mechanics with compliance-guided PEEP and peak pressure settings as follows:

First, PEEP level was determined by an incremental (or decremental) PEEP titration, which represents the point of the highest tidal volume or consecutive dynamic compliance at a fixed driving pressure. Subsequently, driving pressure was stepwise increased, as long as tidal volume showed an overproportional increase based on measured dynamic compliance. This strategy allows for a precise determination of the (so-called) lower and upper inflection point during ventilation and leads to an almost linear relationship of pressure and volume during the ventilation cycle within the individual limits of dynamic lung mechanics [8] (Fig. 3a + b). Finally, gas flow was set to achieve a paCO_2_ level < 60 mmHg (8.0 kPa).

Calculation of alveolar pressure according to Ohm’s law revealed a P_peak_ of 21 cm H_2_O and a PEEP of 9 cm H_2_O, thus a driving pressure of 12 cm H_2_O, resulting in a compliance of 27 ml/cm H_2_O and a calculated MP [9] of 8.6 J/min with individualized ventilation settings. Thirty minutes after compliance-guided adjustment paO_2_/FiO_2_ ratio was 140 (Fig. 2b).

Re-evaluation of lung mechanics after two hours allowed for a further reduction of P_peak_ to 20 cm H_2_O and a PEEP of 7 cm H_2_O (Fig. 2c) resulting in a MP of 7.7 J/min. However, during a period of four hours after initial assessment of the patient’s lung mechanics, the compliance worsened, and subsequently tidal volume decreased from 5.3 to 4.7 ml/kg PBW with a decline of paO_2_ to 67.6 mmHg (9.0 kPa) at an FiO_2_ of 0.65 (paO_2_/FiO_2_ ratio of 104).

Ventilation of the patient was then continued with PCV at significantly increased pressure settings (P_peak_ 28 cm H_2_O, PEEP 10 cm H_2_O, respiratory rate 25 /min, MP 17.3 J/min) and the patient was put in prone position. In the further course ongoing invasive ventilation led to ECMO therapy and unfortunately the patient died two months later due to therapy refractory B-cell depletion and no ability to form antibodies against SARS-CoV-2.

## Discussion

FCV enables a continuous, constant flow not only during inspiration, but also during expiration, which is a novelty in artificial ventilation [10, 11]. Steady, slow changes in pressure and volume minimize the mechanical impact of ventilation (i.e. stress and strain) or – in terms of physics – mechanical power and dissipated energy. Previous preclinical [12, 13] as well as clinical trials [14–16] with FCV already demonstrated improved lung recruitment and gas distribution, which was hypothesized to be related to flow control and a linearized airway pressure decline during expiration. In our case, this may explain the initial increase in paO_2_/FiO_2_ ratio by 30 % (from 130 to 183) with identical ventilation settings as in preceding PCV.

Strictly speaking, due to physical reasons, pressure settings were not the same in PCV and FCV. The continuous, constant flow in FCV results in a pressure drop from the trachea to the alveolar space during inspiration and reversely during expiration. The pressure measured in the trachea and shown on the monitor of the ventilator therefore does not reflect the “true” (= effective) alveolar pressure, which according to Ohm’s law must be lower during inspiration and higher during expiration, given that gas flow and resistance of the system are kept constant. This specific physical characteristic must be kept in mind when assessing pressure settings during ventilation with FCV and thus higher driving pressures and lower PEEP levels than generally accepted in PCV [17–20] may be applicable in FCV.

The only uncertainty is the amount of tissue resistance (resulting from e.g. inertia, friction, viscoelasticity) not contributing to the flow-related difference between tracheal and alveolar pressures. In FCV constant in- and expiratory flow allows for more precise resistance measurements at P_peak_ (representing airway as well as tissue resistance) as well as PEEP (mainly representing airway resistance) and a well-founded estimation of tissue resistance (as the difference of both values). To keep it simple, in our patient only the resistance measured at P_peak_ level was used for conversion of tracheal pressures into (global) alveolar pressures.

Compliance-guided pressure settings aim to reduce both the occurrence of atelectasis and overdistension of lung tissue by adjusting the patient’s ventilation between the (so-called) upper and lower inflection point of the individual pressure-volume curve (Fig. 2a + b). Because in FCV the degree of freedom is limited to settings of P_peak_, PEEP and gas flow, the administered tidal volume and respiratory rate will automatically adjust depending on the amount of functional lung tissue. This fact is of high importance especially in COVID-19 patients, as they can show quick alterations in lung compliance.

An issue to be discussed is the low PEEP level determined during compliance-guided pressure adjustments, which apparently did not fit the demands of our ARDS patient. Initial rapid improvement of gas exchange after switching from PCV to FCV was probably related to a slight increase in PEEP (with the same PEEP set at the ventilator as during PCV) due to the nature of FCV as explained above. However, two hours later PEEP was decreased to 5 cm H_2_O (corresponding to a PEEP of approximately 7 cm H_2_O in the alveolar space) after re-evaluation of the patient. Finally, the patient’s very poor compliance (< 20 ml/cm H_2_O) and significantly reduced PEEP level (5 cm H_2_O) resulted in a tidal volume not capable of maintaining sufficient oxygenation (4.7 ml/kg PBW). This fact could have been anticipated by examination of the corresponding pressure-volume (PV) loops, which upon detailed analysis indicated alveolar collapse at the end of expiration and subsequent initial alveolar recruitment during inspiration (initial kinking of the inspiratory pressure volume curve, Fig. 3c).

Unfortunately, the PV loop feature is not implemented in the respirator’s software yet which undoubtedly would be very helpful to monitor undesired changes in dynamic lung mechanics in critically ill patients.

## Conclusions

In FCV due to constant flow, monitored tracheal pressure does not reflect the pressure at the alveolar space and thus higher driving pressures and lower PEEP levels than generally accepted may be applicable. Additionally, in FCV individualization of ventilation settings based on dynamic compliance measurements is possible, even though it needs constant reevaluation of the patient’s lung mechanics and a prompt adjustment of ventilator settings if it becomes clear that the severity of the pulmonary disease impedes a lung-protective ventilation strategy. Under these circumstances increased driving pressures or PEEP levels above lung mechanic limits and/or an increased fraction of inspired oxygen must be accepted. This case may demonstrate the limits of a lung-protective ventilation strategy in ARDS and highlights the complexity of this disease. However, FCV might offer a clinical applicable approach to reduce applied and dissipated energy of artificial ventilation in order to improve the outcome in COVID-19 patients requiring prolonged ventilation.

## Data Availability

The datasets used and analyzed are available from the corresponding author on reasonable request.

## Abbreviations

ARDS: Acute repiratory distress syndrome
COVID-19: Corona virus disease 2019
CT: Computed tomography
ECMO: Extracorporeal membrane oxygenation
ELSO: Extracorporeal life support organisation
FCV: Flow-controlled ventilation
FiO2: Fraction of inspired oxygen
I:E: Inspiration to expiration ratio
MP: Mechanical power
paCO_2_: Arterial partial pressure of carbon dioxide
paO_2_: Arterial partial pressure of oxygen
PBW: Predicted body weight
PCV: Pressure controlled ventilation
PEEP: Positive end-expiratory pressure
PV Loop: Pressure volume loop
R: Resistance
$$ \dot{V} $$: Flow
ΔP: Pressure difference
